# Future therapeutic targets in rheumatoid arthritis?

**DOI:** 10.1007/s00281-017-0623-3

**Published:** 2017-04-27

**Authors:** Tommy Tsang Cheung, Iain B. McInnes

**Affiliations:** 1 Department of Medicine, Li Ka Shing Faculty of Medicine, Queen Mary Hospital, The University of Hong Kong, 102 Pokfulam Road, Hong Kong, Hong Kong; 2 Institute of Infection, Immunity and Inflammation, University of Glasgow, B4/13, GBRC, 120 University Place, Glasgow, G12 8TA UK

**Keywords:** Rheumatoid arthritis, GM-CSF, JAK, BTK, PI3K, Tolerogenic dendritic cells

## Abstract

Rheumatoid arthritis (RA) is a chronic inflammatory disease characterized by persistent joint inflammation. Without adequate treatment, patients with RA will develop joint deformity and progressive functional impairment. With the implementation of treat-to-target strategies and availability of biologic therapies, the outcomes for patients with RA have significantly improved. However, the unmet need in the treatment of RA remains high as some patients do not respond sufficiently to the currently available agents, remission is not always achieved and refractory disease is not uncommon. With better understanding of the pathophysiology of RA, new therapeutic approaches are emerging. Apart from more selective Janus kinase inhibition, there is a great interest in the granulocyte macrophage-colony stimulating factor pathway, Bruton’s tyrosine kinase pathway, phosphoinositide-3-kinase pathway, neural stimulation and dendritic cell-based therapeutics. In this review, we will discuss the therapeutic potential of these novel approaches.

## Introduction

Rheumatoid arthritis is a chronic inflammatory disease characterized by persistent joint inflammation. Without adequate treatment, patients with rheumatoid arthritis (RA) will develop joint deformity and progressive functional impairment. Substantial evidence indicates that persistent systemic inflammation and immune dysfunction plays a major role in the development of co-morbidities, such as cardiovascular diseases, osteoporosis, interstitial lung disease and malignancies. Large retrospective cohorts have shown that the risk of myocardial infarction is at least 1.5 times higher compared with controls [1, 2] and patients with RA have increased cardiovascular mortality as a result [3–5]. In addition, many studies consistently indicate an increase in the incidence of malignancies, such as lymphoma [6–8]. As a result, patients with RA have reduced quality of life and life expectancy.

With the implementation of treat-to-target strategies, the outcomes of patients with RA have significantly improved. The likelihoods of achieving remission and low disease activity are significantly higher compared with usual care and historical controls. As a result, those patients experience less functional impairment [9–15]. Strategic approaches of this nature not only alleviate clinical symptoms of RA but also demonstrate significant benefits to RA-associated co-morbidities. Osteoporosis is significantly less frequent in patients with disease remission, and a similar trend was also observed for cardiovascular disease [16]. Patients in remission have a significant reduction in cardiovascular risk that is comparable to that of healthy controls [17].

In parallel, biologic therapies have revolutionized the treatment paradigm of RA because they are generally more effective than conventional synthetic disease-modifying anti-rheumatic drugs (csDMARDs). Even biologic therapies currently available only demonstrate clinical efficacy in about two thirds of patients. As a result, the unmet need in the treatment of RA remains high, remission rates are insufficient and new therapeutic approaches should be explored especially for those patients with refractory disease. In this review, we will discuss the potentials of several novel therapeutic agents.

## Extracellular target in RA

A range of extracellular targets are currently under consideration. The majority concern previously targeted cytokines, e.g. IL-6R or ligands, IL-6. Recent studies targeting a variety of cytokines, e.g. IL-17, IL-20 and IL-21, have been disappointing [18–24]. Herein, we will focus on one novel cytokine that has elicited promising data in early trials.

### Granulocyte macrophage-colony stimulating factor

Granulocyte macrophage-colony stimulating factor (GM-CSF) is a haematopoietic growth factor responsible for the differentiation and proliferation of myeloid cells, including neutrophils, dendritic cells and macrophages. In addition, GM-CSF also induces migration and proliferation of endothelial cells [25]. It is produced by a wide variety of cell types, such as myeloid cells, lymphocytes and tissue-resident cells including chondrocytes, fibroblasts, osteoblast and endothelial cells [26, 27]. Production of GM-CSF can be stimulated by multiple agents, such as lipopolysaccharide, tumour necrosis factor, IL-1 and IL-23 [28]. It binds to a heterodimeric GM-CSF receptor, which consists of a ligand-specific binding α-chain and a signal transducing β-chain [29]. Subsequent signalling from the GM-CSF receptor activates Janus kinase-signal transducer and activator of transcription (JAK-STAT), phosphoinositide-3-kinase (PI3K) and MAPK pathway [30, 31].

GM-CSF plays a crucial role in innate immune responses. In general, it enhances the effector functions of neutrophils and macrophages, leading to increased expression of adhesion molecules, production of inflammatory cytokines and activation of phagocytosis [32]. GM-CSF can also polarize macrophages into an inflammatory M1 phenotype, which are involved in synovial inflammation [33]. GM-CSF is also involved in the development, maturation, antigen presentation and cytokine production by dendritic cells [34–36]. Several in vitro studies show that GM-CSF stimulates the development of inflammatory dendritic cells. These inflammatory dendritic cells produce pro-inflammatory cytokines, such as TNF, IL-12 and IL-23. Following antigen engagement and conditioning by the pro-inflammatory cytokines, the inflammatory dendritic cells are able to present self-antigens and stimulate T cell in the lymph nodes [37, 38].

In patients with RA, GM-CSF is expressed in the synovial membrane and the level of GM-CSF is increased in the synovial fluid [39]. Increased expression of GM-CSF receptors is found on circulating monocytes [40], which promotes the subsequent maturation and activation of macrophages in the synovium. In addition, GM-CSF receptors are up-regulated in the synovial tissue [41], and in vitro studies show that GM-CSF induces the proliferation of fibroblast-like synoviocytes [42]. GM-CSF also contributes to the differentiation and survival of Th17 cells and dendritic cells [43]. In the presence of GM-CSF, monocyte-derived dendritic cells maintain their inflammatory potential and are resistant to the immunosuppressive effect of IL-10 in vitro [44]. These dendritic cells also produce high levels of IL-1, IL-6 and TNF-α [45] and are able to present auto-antigens via MHC molecules, contributing to the pathogenesis of RA [46].

Based on these findings, GM-CSF inhibition is an attractive therapeutic target for RA. In animal models, administration of GM-CSF exacerbates arthritis in the collagen-induced arthritis (CIA) model, while administration of neutralizing antibodies against GM-CSF prevents disease progression [47, 48]. In view of the robust effects of GM-CSF inhibition demonstrated in pre-clinical studies, the efficacy of GM-CSF inhibitors has been evaluated in clinical trials [49].

Mavrilimumab is a monoclonal antibody against the human GM-CSF receptor alpha chain. In the EARTH EXPLORER I study, 326 patients with active RA and who had previously an inadequate response to DMARDs were randomized to receive different doses of mavrilimumab versus placebo. In this phase IIb study, a greater proportion of patients receiving mavrilimumab achieved a ≥1.2 decrease in disease activity score (DAS)28-CRP from baseline and the 100-mg dose demonstrated a significant effect versus placebo (23.1 vs. 6.7%). Besides, patients receiving mavrilimumab achieved higher American College of Rheumatology (ACR) response rates at week 24 (ACR20—69.2 vs. 40.0%, *p* = 0.005; ACR50—30.8 vs. 12.0%, *p* = 0.021; ACR70—17.9 vs. 4.0%, *p* = 0.030). Adverse events were generally mild or moderate in intensity. No significant hypersensitivity reactions, serious or opportunistic infections, or changes in pulmonary parameters were observed [50]. In the EARTH EXPLORER II study, 138 patients with active RA and had an inadequate response to tumour necrosis factor inhibitors were randomized to receive mavrilimumab 100 mg every 2 weeks or golimumab 50 mg every 4 weeks for 24 weeks. The difference in the numbers of patients achieving ACR20 response was not statistically different between groups (65.6 vs. 62%, *p* = 0.666) (NCT01715896). As a result, further large-scale studies are warranted to confirm the therapeutic potential of mavrilimumab and to determine its optimal position in the treatment algorithm of RA.

## Intracellular targets in RA

### Janus kinase-signal transducer and activator of transcription pathway

JAK is a receptor tyrosine kinase that mediates intracellular signalling through the transcription factor STAT. In humans, the JAK-STAT pathway is the principal signalling cascade for a wide variety of cytokines and growth factors [51–54]. Intracellular activation of JAK occurs when ligand binding induces the crosslinking of receptor subunits. Activated JAKs phosphorylate the tyrosine residues on the receptors, allowing the binding of STATs in the SRC2 homology domain. JAK then phosphorylates the tyrosine residues on the STATs, leading to dimerization and activation of the STATs. Activated STATs then migrate via the cytoplasm to translocate into the nucleus, where they induce transcription of target genes [54].

The JAK family comprises four members, JAK1, JAK2, JAK3 and Tyk2. JAK1, JAK2 and Tyk2 are expressed ubiquitously, while JAK3 has limited expression in haematopoietic cells [55]. JAK1 interacts with a wide variety of cytokines, through the common γ chain receptor subunit (IL-2 and IL-4 receptor family) and the glycoprotein-130 subunit. JAK1 is also involved in type 1 interferon signal transduction. JAK2 also interacts with cytokines through the glycoprotein-130 subunits, as well as other hormones including erythropoietin, thrombopoietin, prolactin and growth hormone. As a result, the use of JAK2 inhibitors may be associated with anaemia and thrombocytopenia. JAK3 interacts with many inflammatory cytokines through the common γ chain receptor subunit [56], and inactivating mutations in the common γ chain and JAK3 have been shown to cause X-linked severe combined immunodeficiency [57]. Patients with X-linked severe combined immunodeficiency have a significantly impaired adaptive immune system due to the absence T cells and non-functional B cells (Table 1).

**Table 1:** Cytokines and hormones activating the JAK pathway

JAK1	JAK2	JAK3
*Common γ chain receptor family* IL-2, IL-4, IL-7, IL-9, IL-15, IL-21		*Common γ chain receptor family* IL-2, IL-4, IL-7, IL-9, IL-15, IL-21
*Gp130 receptor family* IL-6, IL-11, IL-27, IL-31	*Gp130 receptor family* IL-6, IL-11, IL-27, IL-31	
*Interferon* IFNα/β/γ	*Interferon* IFNα/β/γ	
	*Hormones* Erythropoietin Thrombopoietin Prolactin Growth hormone	
	*GM-CSF receptor family* IL-3R, IL-5R, GM-CSF-R	

Many JAK inhibitors have been studied for the treatment of RA [58]. Tofacitinib, the first-in-class JAK inhibitor, has been shown to be efficacious in different clinical settings—it blocks JAK1 and JAK3 preferentially [59–64] (Table 2). In the ORAL standard trial, patients with an inadequate response to methotrexate were randomized to receive tofacitinib, adalimumab or placebo plus background methotrexate. At 6 months, patients receiving tofacitinib (5 or 10 mg twice daily) and adalimumab 40 mg every 2 weeks achieved higher ACR20 response rates (51.5, 52.6 and 47.2%, respectively), compared with those receiving placebo (28.3%). There were also greater reductions in the Health Assessment Questionnaire Disability Index (HAQ-DI) scores at 3 months and higher percentages of patients achieving DAS remission at 6 months [62]. In the ORAL scan trial, patients receiving tofacitinib (5 or 10 mg twice daily) had less radiological progression. The mean changes in total modified Sharp score for tofacitinib at 5 and 10 mg twice daily were 0.12 and 0.06, respectively, versus 0.47 for placebo [60]. Patients with inadequate response to tumour necrosis factor inhibitors were evaluated in the ORAL step trial. The addition of tofacitinib (5 or 10 mg twice daily) to methotrexate (MTX), compared with placebo plus MTX, resulted in significantly higher ACR20 response rates (42, 48 vs. 24%, respectively) and greater reductions in the HAQ-DI scores at 3 months [61].

**Table 2:** Clinical trials of tofacitinib

Study name	Number of subjects	Subject characteristics	Intervention	Primary endpoints	Results
ORAL start [59]	958	MTX naive	Tofacitinib (5 or 10 mg) vs. MTX 20 mg per week	ACR70 response Mean change in modified total Sharp score	25.5, 37.7 vs. 12.0% 0.2, <0.1 vs. 0.8
ORAL scan [60]	797	MTX-IR	Tofacitinib (5 or 10 mg) vs. placebo	ACR20 response Mean change in modified total Sharp score Mean change in HAQ-DI DAS remission	51.5, 61.8 vs. 25.3% 0.12, 0.06 vs. 0.47^a^ −0.40, −0.54 vs. −0.15 7.2, 16 vs. 1.6%
ORAL solo [63]	611	DMARD-IR*	Tofacitinib (5 or 10 mg) vs. placebo	ACR20 response Mean change in HAQ-DI DAS remission	59.8, 65.7 vs. 26.7% −0.50, −0.57 vs. −0.19 5.6, 8.7 vs. 4.4% (NS)
ORAL sync [64]	792	DMARD-IR*	Tofacitinib (5 or 10 mg) vs. placebo	ACR20 response Mean change in HAQ-DI DAS remission	52.1, 56.6 vs. 30.8% -0.44, -0.53 vs. -0.16 8.5, 12.5 vs. 2.6%
ORAL standard [62]	717	MTX-IR	Tofacitinib (5 or 10 mg) vs. adalimumab 40 mg q2w vs. placebo	ACR20 response Mean change in HAQ-DI DAS remission	51.5, 52.6, 47.2 vs. 28.3% 0.55, −0.61, 0.49 vs. −0.24 6.2, 12.5, 6.7 vs. 1.1%
ORAL step [61]	399	TNFi-IR	Tofacitinib (5 or 10 mg) vs. placebo	ACR20 response Mean change in HAQ-DI DAS remission	41.7, 48.1 vs. 24.4% −0.43, −0.46 vs. −0.18 6.7, 8.8 vs. 1.7%

*At least 1 non-biologic or biologic DMARD#262

^a^Since tofacitinib 5 mg twice daily failed to show statistically significance for radiographic progression, and due to the step-down procedure applied to the primary efficacy endpoints, significance was not declared for the HAQ-DI and DAS remission

The relative safety of tofacitinib has generally appeared similar to that of biologic DMARDs, including increased risk of infections and liver function derangements, cytopenias, hyperlipidaemia and, possibly, increased serum creatinine levels. Gastrointestinal perforations have also been reported. The incidence was similar to the published data of tumour necrosis factor inhibitors and perhaps lower than that associated with tocilizumab. It has been postulated that this potential side effect could be due to a significant inhibition of IL-6 production. The risk of herpes zoster reactivation was found to be significantly increased in patients receiving tofacitinib. Data from the phase II, III and long-term extension studies showed that the incidence rate of herpes zoster reactivation was 4.4 per 100 patient-years in patients receiving tofacitinib, compared to 1.5 per patient-years in the placebo arm. The risk is substantially higher among Asians, the elderly and those using higher doses of glucocorticoids [65].

Baricitinib, another novel JAK inhibitor, has also been evaluated in several phase III clinical trials. It preferentially inhibits JAK1 and JAK2 and demonstrates efficacy in patients with RA in different clinical settings [66–69] (Table 3). In the RA-BEAM trial, patients with active RA on background MTX were randomized to receive baricitinib 4 mg daily, adalimumab 40 mg every 2 weeks or placebo (switched to baricitinib at week 24) for 52 weeks. At week 12, patients receiving baricitinib were more likely to achieve ACR20 response compared with those receiving adalimumab or placebo (70 vs. 61 vs. 40%). The ACR20 response rates at week 12 in patients receiving 4 mg baricitinib and placebo were 70 and 40%, respectively. There were also greater reductions in the HAQ-DI scores and progression of mean total modified Sharp scores at 24 weeks [69]. Similar results were also demonstrated in the RA-BUILD trial. Patients with active RA and who had an inadequate response to conventional synthetic DMARDs were randomized to receive baricitinib (2 or 4 mg daily) or placebo. Patients receiving 4 mg baricitinib achieved higher ACR20 response rates than those receiving placebo at 12 weeks [66]. In addition, patients who had an inadequate response to tumour necrosis factor inhibitors also showed a significant improvement after receiving high-dose baricitinib. The ACR20 response rates at week 12 in patients receiving 4 mg baricitinib and placebo were 55 and 27%, respectively [67]. To date, baricitinib is pending approval by the FDA and EMA.

**Table 3:** Clinical trial of baricitinib

Study name	Number of subjects	Subject characteristics	Intervention	Primary endpoints	Results
RA-BEGIN [68]	588	DMARD naive	Baricitinib 4 mg + MTX 10-20 mg per week vs. baricitinib 4 mg vs. MTX 10–20 mg per week	ACR20 response	77 vs. 62%^a^
RA-BEAM [69]	1307	MTX-IR	Baricitinib 4 mg vs. adalimumab 40 mg q2w vs. placebo	ACR20 response	70 vs. 61 vs. 40%^b^
RA-BUILD [66]	684	DMARD-IR	Baricitinib (2 or 4 mg) vs. placebo	ACR20 response	62 vs. 39%^c^
RA-BEACON [67]	527	TNFi-IR	Baricitinib (2 or 4 mg) vs. placebo	ACR20 response	55 vs. 27%^c^
RA-BEYOND	Estimated 3073		Baricitinib (2 or 4 mg)	1 drug-related adverse event or any serious adverse events	Ongoing

^a^The primary endpoint is a non-inferiority comparison of baricitinib monotherapy to MTX monotherapy

^b^The primary endpoint is a superiority comparison of baricitinib therapy to placebo. Baricitinib therapy is compared with adalimumab based on non-inferiority design

^c^The primary endpoint is the comparison between baricitinib 4mg and placebo

Meanwhile, other JAK inhibitors are being evaluated in phase III clinical trials. Filgotinib developed by Galapagos NV/Gilead and ABT-494 by AbbVie are both selective JAK1 inhibitors. The use of these agents can theoretically reduce the risk of JAK2- and JAK3-associated adverse reactions. Both filgotinib and ABT-494 showed promising results in patients with RA in phase IIb studies [70–73], and phase III studies are being conducted to evaluate their therapeutic efficacies (Table 4).

**Table 4:** Clinical trials of filgotinib and ABT-494

Study drug	Estimated enrolment	Subject characteristics	Intervention	Primary endpoints	ClinivalTrials.gov identifier
Filgotinib	1200	MTX naive	Filgotinib + MTX vs. filgotinib vs. MTX	ACR20 response	NCT02886728
	1650	MTX-IR	Filgotinib vs. adalimumab vs. placebo	ACR20 response	NCT02889796
	423	bDMARD-IR	Filgotinib vs. placebo	ACR20 response	NCT02873936
ABT-494	975	MTX naive	ABT-494 vs. MTX	ACR50 response; DAS28-CRP remission	NCT02706873
	1500	MTX-IR	ABT-494 vs. adalimumab vs. placebo	ACR20 response	NCT02629159
	600	MTX-IR	ABT-494 vs. MTX	ACR20 response; DAS28-CRP LDA	NCT02706951
	600	csDMARD-IR	ABT-494 vs. placebo	ACR20 response; DAS28-CRP LDA	NCT02675426

### Bruton’s tyrosine kinase pathway

Bruton’s tyrosine kinase (BTK) is another key intracellular kinase being actively investigated for the treatment of RA in addition to other immune-mediated disorders, e.g. SLE. It is a member of the Tec family of non-receptor tyrosine kinases with restricted expression in B cells and myeloid cells, such as macrophages and dendritic cells [74]. It plays a crucial role in B cell development and activation. When an antigen binds to the B cell receptor, spleen tyrosine kinase (SYK) is activated and leads to subsequent phosphorylation of adaptor proteins recruiting PI3K to the plasma membrane. As a result, intracellular levels of phosphatidylinositol-3,4,5-triphosphate (PIP3) increases [75]. PIP3 binds to the pleckstrin homology domain of BTK and thereby promotes the recruitment of BTK to the plasma membrane [76]. Subsequent phosphorylation of BTK by the Src-kinases and SYK activates phospholipase C-γ2, which leads to nuclear factor κB and nuclear factor of activated T cells transcriptional activation [77]. Through these signalling pathways, BTK activation induces B cell survival, proliferation and differentiation into plasma cell. Mutations of the BTK genes are associated with the development of X-linked agammaglobulinaemia in human and X-linked immunodeficiency in mice [78, 79]. In addition to B cell receptor signalling, BTK is also associated with macrophage signalling through the FCγ receptors. In the absence of BTK, FCγ receptor-associated functions are impaired. In animal models, macrophages from mice with X-linked immunodeficiency produce less nitric oxide, TNF-α and IL-Iβ [80, 81].

Given that BTK is functionally active in both B cells and myeloid cell function, it is an attractive therapeutic target for RA. In particular, inhibiting the kinase activity of BTK would block multiple signalling pathways in different cell populations. In the CIA model, mice with mutations in BTK have decreased susceptibility to develop arthritis [82]. Similarly, FCγ receptor-deficient mice do not develop arthritis, even with the presence of auto-antibodies [83]. Consequently, several small molecule BTK inhibitors have been developed and demonstrated efficacy in animal models of RA [84]. However, only six BTK inhibitors are currently reported to be in clinical development for RA. All of the inhibitors selectively target the ATP-binding pocket of BTK, and the irreversible inhibitors covalently bind the cysteine residue in the active site of the kinase domain [85].

CC-292 is the first irreversible BTK inhibitor that has been evaluated in a phase II study. Forty-seven patients with active RA and had an inadequate response to methotrexate were randomized to receive either CC-292 375 mg daily versus placebo. The primary endpoint was the ACR20 response at 4 weeks. The study was completed in March 2016 but the results have not been published so far (NCT01975610). Other potential BTK inhibitors are still being investigated in pre-clinical or early phase clinical studies. The phase I study on HM71224 (NCT01765478), developed by Hamni, has just been completed, while the phase I study on TK-020 (NCT02413255), developed by Takeda, is still ongoing [86].

### Phosphoinositide-3-kinase pathway

PI3Ks are lipid-signalling kinases that phosphorylate phosphoinositides to produce phosphorylated inositol lipids. PI3Ks are classified into class I, II and III according to their structures, regulations and lipid substrate specificities [87, 88]. To date, only the functions of class I PI3Ks have been well characterized, whereas the roles of class II and III PI3Ks in humans are yet to be elucidated. Class I PI3Ks, which include PI3Kα, PI3Kβ, PI3Kδ and PI3Kγ, catalyze the phosphorylation of phosphatidylinositol-4,5-bisphosphate to phosphatidylinositol-3,4,5-trisphosphate (Table 5). Through subsequent signalling cascades, class I PI3Ks control many cellular functions, such as growth, proliferation, survival and apoptosis, as well as leukocyte adhesion and migration. PI3Kα and PI3Kβ are ubiquitously expressed, whereas PI3Kδ and PI3Kγ are preferentially expressed in leukocytes and play a crucial role in innate and adaptive immune response.

**Table 5:** Isoforms of PI3K

	PI3K isoform	Catalytic subunit	Regulatory subunits	Substrate	Product
Class IA	PI3Kα PI3Kβ PI3Kδ	p110α p110β p110δ	p85α, p85β, p55α, p55γ, p50α	PI-4,5-P2	PIP3
Class IB	PI3Kγ	p110γ	p101, p84	PI-4,5-P2	PIP3
Class II		PIK3-C2 PIK3-C3 PIK3-C2		PI-4-P PI	PI-3,4P2 PI-3-P
Class III		VPS34	P150	PI	PI-3-P

In T cells, class I PI3Ks are activated by T cell receptor (TCR) or IL-2 receptor engagement. In p110γ knockout T cells, TCR-mediated early signalling is relatively unaffected, but they proliferate less after stimulation with CD3-specific antibody and produce lower levels of cytokines in vitro [89]. Similarly, PI3Kδ kinase inactive knockin mice also show reduced T cell proliferation and activation in vitro, and impaired antigen-specific T cell responses in vivo [90]. In addition, PI3Kδ is involved in the differentiation of T cells. In animal models, p110δ mutant mice had impaired differentiation along the T helper (Th) 1 and Th 2 lineage [91] and exhibited a significant decrease in T helper 2 cytokine responses [92]. P110δ mutant mice also had reduced regulatory T cell (Treg) populations in the spleen and lymph nodes, implicating PI3Kδ in the maintenance of Treg in the periphery. Moreover, Treg cells with inactive PI3Kδ had attenuated secretion of IL-10 to suppress T cell proliferation in vitro [93].

PI3Kδ is essential for B cell development and function as it generates survival signals even without antigen binding to the B cell receptors [94–96]. In animal models, PI3Kδ mutant mice have significant reductions in the IgM producing B cell and marginal zone B cell populations [96]. B cell proliferative responses to IL-4 stimulation and T cell-independent antibody responses in PI3Kδ mutant mice are also attenuated [97, 98]. Furthermore, B cells deficient in p110δ showed diminished chemotactic responses to CXCL13 and CXCR5-dependent B cell homing to Peyer’s patches [99].

In common with other leucocytes, class I PI3Ks are markedly enriched in neutrophils. In p110γ knockout mice, neutrophils migrate less efficiently towards chemokines and chemo-attractants, such as complement C5a and bacterial peptide N-formyl-methionyl-leucyl-pehnylalamine (fMLP) [100, 101]. Neutrophils with inactive p110γ also demonstrated impaired reactive oxygen species production [101] and neutrophil respiratory burst in response to fMLP [102]. Apart from neutrophils, dendritic cells from p110γ knockout mice also developed impaired migration to the site of inflammation [103]. Although the involvement of PI3Kδ in neutrophil functions has been controversial, PI3Kδ may have a role in promoting IL-6 release in response to cKit stimulation on dendritic cells [104].

Based on these findings, PI3Kδ and PI3Kγ have attracted considerable interest as pharmacological targets in the treatment of RA [105, 106]. In the CIA model, mice treated with a p110γ selective inhibitor show significant reduction in joint inflammation. Histological measures of synovial inflammation and neutrophil infiltration in arthritic joints are also significantly attenuated [107]. PI3Kγ is also implicated in the regulation of synovial fibroblasts, in which p110γ deficiency leads to a milder inflammatory-erosive arthritis and TNF-mediated cartilage destruction through reduced expression of matrix metalloproteinases in fibroblasts and chondrocytes. In vitro analyses confirmed that the decreased invasiveness of fibroblasts is mediated by reduced phosphorylation of Akt and extracellular signal-regulated kinase. Similar findings using a PI3Kγ-specific inhibitor in human synovial fibroblasts from patients with RA who exhibit disease-specific up-regulation of PI3Kγ was also confirmed in this study [108]. A recent study also reported the role of PI3K signalling pathway in synovial angiogenesis in the CIA model. Hypoxia-inducible factor 1α and vascular endothelial growth factor are up-regulated through PI3K signalling and mediates subsequent neovascularization of the synovium [109].

At present, PI3K inhibition is a major focus in the field of oncology. Idelalisib, the first-in-class PI3K inhibitor, appears efficacious in refractory chronic lymphocytic leukaemia and indolent non-Hodgkin’s lymphoma and has provided some safety data concerning PI3K inhibition [110–113]. Many pharmaceutical companies are exploring the potential of PI3K inhibitors in autoimmune diseases, like RA. However, no PI3K inhibitor has yet entered a clinical development programme to our knowledge.

## Neuropathways in RA

The reciprocal effects of the nervous system on immunity has raised recent interest. It is well known that the nervous system regulates inflammation through peripheral nerves and a variety of neurotransmitters and neuropeptides. In animal models, peripheral denervation attenuates joint inflammation in mice with adjuvant induced arthritis [114]. An imbalance of the autonomic nervous system has been implicated in many inflammatory conditions. In general, activation of para-sympathetic nervous system mediates an anti-inflammatory response, while the sympathetic nervous system may have both pro-inflammatory and anti-inflammatory properties [115]. The para-sympathetic nervous system, through the vagus nerve, exerts anti-inflammatory actions. In a mouse model, electrical stimulation of the peripheral part of the vagus nerve significantly decreases serum TNF-α levels in rats with bilateral cervical vagotomy. In vitro studies showed that acetylcholine (ACh) inhibits pro-inflammatory cytokine release by the macrophages in a dose dependent fashion [116]. Subsequent research had identified the neuronal type α7-ACh receptor subtype as the essential regulator of the anti-inflammatory effects mediated by the para-sympathetic nervous system [117]. α7-ACh receptors are not only found on neurons but also widely expressed in immune cells and fibroblast like synoviocytes [118–120]. In the CIA model, administration of a specific α7-ACh receptor agonist showed effective attenuation of arthritis and systemic inflammatory responses [121, 122]. Conversely, α7-ACh receptor knockout mice developed more severe arthritis compared with the wild type controls [123]. Apart from the effects mediated by immune cells, activation of the α7-ACh receptor in the fibroblast like synoviocytes also suppresses the production of pro-inflammatory cytokines [119, 124]. The spleen also plays an important role in the regulation of systemic inflammation. Although there is no evidence showing that lymphoid organs are directly innervated by the para-sympathetic innervation, studies have shown that the spleen is able to receive signals from the para-sympathetic system and vagus nerve activation may inhibit TNF-α production by splenic macrophages via the celiac superior mesenteric plexus [125, 126]. Recently a clinical trial provided proof of concept that vagal nerve stimulation could be therapeutically feasible in RA [127]. In this study, 17 patients with RA received vagus nerve stimulation. At day 42, DAS28-CRP levels significantly improved from baseline (6.05 ± 0.18 vs. 4.16) and the results were more robust in biologic naive patients. The proportions of patients achieving ACR20, ACR50 and ACR70 response were 71.4, 57.1 and 28.6%, respectively. Among patients who responded to vagus nerve stimulation, their serum IL-6 levels were significantly reduced from baseline and the reduction in IL-6 levels correlated with the improvement in disease activity [127].

The sympathetic nervous system, in turn, mediates its effect on the immune system via catecholamine production. Both primary and secondary lymphoid organs are innervated by the sympathetic nervous system [128, 129]. Lymphocytes primarily express the β2 adrenergic receptors while cells of the innate immune systems express α1, α2 and β2 adrenergic receptors [129]. The effect of sympathetic nervous system on the immune system is more complex. In general, sympathetic activation is able to inhibit the development of a Th1 immune response [128]. Patients with RA have an imbalance of the autonomic nervous system, with increased sympathetic and reduced para-sympathetic activities [130]. However, peripheral mononuclear cells from patients with RA express lower levels of β2 adrenergic receptors and, therefore, less effective in suppressing T cell activation and proliferation via β2 adrenergic activation [131]. In contrary, catecholamines mediate their effects through α1 adrenergic receptors on the peripheral mononuclear cells [132], leading to increased production of IL-6 [133]. In addition, α2 stimulation promotes the proliferation of fibroblast-like synoviocytes in patients with RA and subsequent production of pro-inflammatory cytokines [134]. At present, modulations of the sympathetic nervous system yield inconsistent effects in animal arthritis models, and none of them has been evaluated in clinical development programme.

## Long-term remission strategies and immune homeostasis

### Dendritic cell therapeutics

Dendritic cells (DCs) play a key role in both the innate and adaptive immunity. In the periphery, DCs exist as immature cells and undergo differentiation after exposure to pro-inflammatory cytokines, immune complexes or pathogens and endogenous inflammatory factors that are recognized by the Toll-like receptors. Mature DCs then migrate to lymph nodes and present antigens on the MHC molecules to the naive T cells. Cytokines produced by the DCs also promote the differentiation and maturation of T and B cells [135]. DCs are important for maintaining intra-thymic and peripheral tolerance. Immature DCs recognize and phagocytose apoptotic cells [136], rendering DCs tolerogenic as they produce immunosuppressive cytokines and promote cross tolerance of the T cells by inducing T cell anergy, clonal deletion or Treg and suppressor T cell differentiation [137–139]. In normal circumstances, this process does not result in DC maturation. However, failed clearance of apoptotic cells or exposure to maturation signals may induce the production of immunogenic DCs [140, 141].

In patients with RA, the synovial DCs are activated in response to pro-inflammatory cytokines, with up-regulation of MHC and co-stimulatory molecule expression [142, 143]. They also produce IL-12 and IL-23 to potentially promote the differentiation of Th1 and Th17 cell [144]. Treatment with TNFi not only ameliorates the clinical symptoms of RA but also reduces the number of activated DCs and inhibits DC maturation [145, 146]. These observations support the strategy of targeting dendritic cells for the treatment of RA.

In CIA models, administration of low doses of semi-mature DCs inhibits disease progression by enhancing Treg populations and suppresses antigen-specific Th1- and Th17-mediated immunity [147]. Treatment of CIA mice with tolerogenic DCs modified by tacrolimus significantly inhibited the severity and progression of disease with the alteration of the proportion of the Th1 and Th17 in the spleen [148]. Similar results have been confirmed by administration of tolerogenic DCs generated by GM-CSF and IL-4 stimulations [149]. These data provide a better understanding and consolidate the role of tolerogenic DCs in the treatment of RA [150, 151].

The first clinical study using tolerogenic DCs was carried out in the University of Queensland. In this phase 1 study, tolerogenic DCs were generated by treatment of monocyte-derived DCs with an inhibitor of NFkB signalling. These tolerogenic DCs are deficient for CD40 expression but express high levels of CD86 [152]. After priming with citrullinated peptide antigens, the tolerogenic DCs were injected intradermally to 18 patients with RA positive for HLA-DR expression. The treatment was well tolerated with no major adverse effects. The results of another phase 1 trial were published recently. It is a randomized, unblinded, placebo-controlled, dose-escalation phase I study. The tolerogenic DCs were generated by pharmacological modulation of monocyte-derived DCs from patients with dexamethasone and vitamin D_3_, together with a Toll-like receptor-4 agonist. The tolerogenic DCs express high levels of MHC class II molecules and intermediate levels of co-stimulatory molecules CD80 and CD86 and produce high levels of IL-10 and TGF-β [150]. Instead of intradermal injection, 13 patients received three different doses of tolerogenic DCs through intra-articular injection under arthroscopic guidance. The primary objective was to assess the safety of intra-articular injection of tolerogenic DCs in patients with RA. No patient developed an exacerbation of arthritis in the target knee within 5 days of treatment. At day 14, arthroscopic synovitis was present in all participants except for one who received 10 × 10^6^ tolerogenic DCs. Two patients receiving 3 × 10^6^ tolerogenic DCs and one patient receiving 10 × 10^6^ tolerogenic DCs demonstrated improvement in vascularity on day 14, whereas no improvement was seen in six patients receiving 1 × 10^6^ tolerogenic DCs or placebo. Synovitis improved in one of three patients in each of the 1 × 10^6^ and 3 × 10^6^ tolerogenic DC cohorts and in both assessable patients in the 10 × 10^6^ tolerogenic DC cohorts. There were no trends in DAS28 or HAQ score or consistent immunomodulatory effects in peripheral blood [153].

Although tolerogenic DC therapy demonstrates promising results in patients with RA, there are some important issues to be tackled for further clinical evaluation. Administration of tolerogenic DC therapy in patients with established autoimmunity may be less efficacious, and therefore, it should perhaps ideally be given to the patient with RA as early as possible. However, the timing of the treatment is still controversial as potentially elevation of regulatory pathways may suffice. Secondly, the selection of auto-antigens for the generation of tolerogenic DCs may be critical for the efficacy of tolerogenic DC therapy in patients with RA. However, these auto-antigens are not always detected and little is known about the immunodominant profile of these auto-antigens in association with RA pathogenesis and disease progression. Besides, careful consideration should be given to the dose of tolerogenic DCs administered because a high dose of tolerogenic DCs may be potentially immunogenic [154–156]. At present, there is no reliable biomarker of tolerance induction that can be measured after administration of tolerogenic DC therapy. Therefore, further studies are necessary for the development of this immunotherapy.

## Conclusion

Recent advances in the understanding of the pathophysiology of RA facilitate the development of new therapeutic agents. Inhibition of the JAK pathway has already been used in clinical practice, and more selective JAK inhibitors are expected to be available in the near future. The therapeutic potential of BTK inhibitors is being evaluated in phase II studies. As a result, it is expected that over time we will see a balance of therapeutic interventions between biologic and small molecule targeted synthetic DMARDs.
